# Maternal Diet Influences Offspring Feeding Behavior and Fearfulness in the Precocial Chicken

**DOI:** 10.1371/journal.pone.0077583

**Published:** 2013-10-29

**Authors:** Nadège Aigueperse, Ludovic Calandreau, Aline Bertin

**Affiliations:** 1 Institut National de la Recherche Agronomique, UMR85, Physiologie de la Reproduction et des Comportements, Nouzilly, France; 2 Centre National de la Recherche Scientifique, UMR7247, Nouzilly, France; 3 Université François Rabelais de Tours, Tours, France; 4 Institut Français du Cheval et de l’Equitation, Nouzilly, France; CNR, Italy

## Abstract

**Background:**

In chicken, oils in the maternal diet confer a specific scent to the yolk. Embryos are known to perceive and memorize chemosensory signals of the surrounding environment; however, the potential impact of the maternal diet has not previously been investigated. In the present study, we hypothesized that chicken embryos memorize the chemical signals of the maternal diet and that this perceptual learning may orient subsequent feeding behavior of the hatchlings.

**Methodology/Principal Findings:**

Laying hens were fed standard food enriched with 2% menhaden oil (MH group) or 2% soybean oil (controls). The scent of menhaden was significantly more detected in MH egg yolks than in control yolks by a human panel. We analyzed the development and behavior of offspring towards different types of food, bearing or not bearing the menhaden scent. When chicks were exposed to a 3-min choice test between the familiar food bearing the menhaden scent and the familiar food without menhaden, no effect of treatment was observed. In a 3-min choice test with unfamiliar food (mashed cereals) MH chicks showed a clear positive orientation toward the unfamiliar food bearing the menhaden scent. By contrast, control chicks showed a preference for the non-odorized unfamiliar food. MH chicks expressed higher emotional reactivity level than control chicks as expressed by food neophobia and longer immobility in a restraint test.

**Conclusion/Significance:**

Chicks exposed *in ovo* to menhaden oil via the maternal diet preferentially oriented their feeding behavior towards food containing menhaden oil, but only when the food was unfamiliar. We propose that oil in the maternal diet engenders maternal effects and contributes to the development of behavioral phenotype in the offspring. *In ovo* chemosensory learning may have evolved to prepare precocial offspring for their environment. This suggests a common principle of embryonic chemosensory learning across vertebrate taxa.

## Introduction

During the last two decades, extensive research has shown that fetuses can respond to and memorize the chemosensory signals to which they are exposed. In mammals, *in utero* odorants – which include metabolites from the mother’s diet – can pass through the placental barrier and enter the amniotic liquid or blood of the fetus [1], [2], [3]. Exposure to such chemosensory signals can orient the behavior of young animals. For example, rabbit offspring exposed *in utero* to cumin preferentially oriented towards this odor; by contrast, rabbit offspring which had not been exposed *in utero* expressed an avoidance behavior [4]. The preferential response of infant mammals to odors or flavors encountered *in utero* and prior to weaning is well documented for lambs [5], dogs [6], rats [7] mice [8] and humans [9], [10], [11].

This positive orientation towards familiar odors influences the feeding behavior of young individuals. Flavors transmitted via the maternal diet have frequently been reported to induce a better acceptance, and even a preference, for food bearing the same flavors [12]. For example, mice offspring exposed *in utero* to *o*-aminoacetophenone–a substance spontaneously aversive for rodents–showed enhanced tolerance to ingestion of water aromatized with this substance [3]. Lambs of mothers fed with food containing oregano during pregnancy ingested greater quantities of food bearing oregano than did lambs of mothers fed with standard food [5]. Human babies were found more willingly to ingest foods bearing flavors of foods eaten by their mothers during pregnancy or lactation [13], [11].

Olfacto-gustatory perceptual learning during the very early developmental stages of non-mammals has been much less frequently investigated. However, pre-imaginal olfactory experiences have been reported for many species of insects, and have been shown to influence a range of behavioral aspects, such as choice of hosts or territory, or even social behavior [14], [15]. The embryos of oviparous species, for example, crocodiles [16], salmon [17] and amphibians are exposed to chemosensory stimuli via the eggshell. After exposure *in ovo* to the scent of orange, the tadpoles of the European common frog (*Rana temporaria*) and the wood frog (*Rana sylvatica*) were found to spend more time in water scented with orange compared to controls. This preference was maintained after metamorphosis in young frogs [18]. In cuttlefish, exposure *in ovo* to the odor of shrimps– the preferred prey– can induce a change of food preference towards crabs [19].

In birds, the influence of maternal diet during egg formation on the behavior of offspring has not previously been investigated. However, in a wide range of avian species, olfaction plays an important role in several behavioral aspects, such as the choice of partner [20], nest construction [21], sexual behavior [22], parental care [23], food gathering [24], [25], spatial orientation [26] [27] and defense against parasites and predators [28], [29]. Moreover, recent studies have demonstrated that bird embryos perceive chemosensory stimuli through the eggshell and, after hatching, exhibit attraction or avoidance towards the familiar stimulus [30], [31], [32], [33]. Józsa et al. [34] showed that the pituitary adenylate cyclase-activating peptide plays a role during *in ovo* olfactory memory formation and subsequent olfactory preferences of chicken.

The aim of the present study was to determine whether the maternal diet of birds can influence the feeding behavior of offspring. We used the domestic chicken as a model, because specific elements, such as fatty acids, in the diet of hens are known to confer a “fishy smell” to the eggs [35]. For example, hens fed with food containing onion, rapeseed oil, or menhaden oil laid eggs for which the odor or savor was qualified as being “similar to the onion” or “fishy” by human panelists [36] [37]. Gas chromatographic/flame photometric detection analysis revealed that volatile flavors in the raw egg yolk of hens fed different diets may be caused by variations in the quantity of sulfur compounds transferred into egg yolk [36]. In the present study, we compared the feeding behaviors of chicks from a group of hens fed a standard food enriched with 2% menhaden oil, and chicks from a group of hens fed a standard food (with equivalent energy content to the enriched food). Fish oils, particularly menhaden oil, are common feed ingredients for laying hens, to increase yolk omega-3 fatty acids [38].

We analyzed the feeding behavior of offspring towards different types of food, bearing or not bearing the menhaden flavor. According to the transnatal chemosensory continuity hypothesis [39], prenatal odor acquisition adaptively guides the behavior of animals in their postnatal niches. Therefore, we expected offspring of hens fed a diet enriched with menhaden oil to show a positive orientation towards foods containing menhaden oil. We expected this attraction to be specific to the prenatal chemosensory stimulus, and non-generalized to other olfactory stimuli [4]. Our study may have applications in the fields of feed engineering and management of parental populations. In the absence of parental care, domestic chicks must learn to identify palatable items from non-palatable items. Establishment of olfactory continuity between the prenatal and post-natal environment via the maternal diet may be a tool to help young birds to identify food items.

## Materials and Methods

### Ethics Statement

All birds were maintained at the Experimental Unit PEAT of INRA (Nouzilly, France). The Experimental Unit is registered by the ministry of Agriculture with the license number B-37-175-1 for animal experimentation. All experiments were approved by the Ethic Committee in Animal Experimentation of Val de Loire CEEA Vdl (permit number 2011-02-8). The CEEA vdl is registered by the National Committe “Comité National de Réflexion Ethique sur l’Expérimentation Animale” under the number 19. All experiments were performed in accordance with the European Communities Council Directive 2010/63/UE.

### Laying Females

#### Housing conditions and feeding treatment

Fourty 20-weeks-old White Leghorn hens (*Gallus gallus domesticus*) from the PEAT experimental unit (INRA, Nouzilly) were split into 2 groups of 20 individuals. The groups were balanced for the mass of the hens, egg mass, and the daily feed intake. Both groups were housed in 2 similar thermo regulated rooms, each with an area of 40 m^2^. Each bird was placed in an individual wire home-pen (100 cm × 100 cm × 50 cm) with wood shavings on the floor, a nest, a perch, a drinker and a trough. In each room, birds had tactile, visual, and vocal contacts with each other. We maintained the hens individually in order to identify the origin of each egg and avoid any influence of agonistic interactions and hierarchy on egg quality. All of the birds were maintained at a temperature of 21±1°C for the duration of the experiment. Water and food were available *ad libitum* during a 14-h light/10-h dark cycle.

After the first week of habituation, during which standard flour was provided as feed, hens were fed with standard granules (unit PEAT, INRA, Nouzilly) for 5 consecutive weeks. The standard granules were supplemented with 2% menhaden oil (Sigma Life Science) for the experimental group and 2% soybean oil for the control group. The 2 types of food were formulated to provide equivalent energy contents (calculated metabolizable energy [ME] = 3100 kcal/kg). Several studies have shown that fish oil, particularly menhaden oil, modifies the chemosensory properties of egg yolk [35]. In the present study, we used 2% of menhaden oil, because this concentration in the diet of Leghorn hens was previously shown to confer a fishy smell to the eggs (as perceived by human panelists) and to have no effect on the egg mass [37].

#### Morpho-physiological measurements

Each hen was weighed 6 times: once during the week before treatment started, and once per week during the 5 weeks of treatment. The daily feed intake was also measured once during the week before treatment started and once per week during the 5 weeks of treatment. The weight of each trough was measured at 24-h intervals, to determine the daily feed intake of each hen.

#### Laying rate and mass of eggs

Eggs from all females were collected and weighed daily for 6 consecutive weeks; during the week before the treatment started, and during the 5 weeks of treatment. The mass of eggs was recorded. The laying rate was calculated as the number of laid eggs per female per day.

#### Tonic immobility test

Intake of omega-3 can increase or decrease the emotional state of mammals [40], [41], we decided to evaluate the emotional reactivity of hens following treatment. Modification of the maternal emotional reactivity may also have an indirect impact on the offspring. For example, in quails (*Coturnix coturnix japonica*), the quality of eggs (mass, yolk hormones) was found to be related to this trait [42], [43]. To evaluate the emotional reactivity of hens we conducted a tonic immobility test during the final week of treatment. In poultry, the duration of tonic immobility is considered to be a standard and robust measure of fearfulness [44]. Each hen was placed on its back in a U-shaped wooden cradle and held by the experimenter with one hand over the sternum and one hand gently covering the bird’s head. The subject was restrained for 10 s prior to release. When more than 10 s had passed between the release and the bird’s escape, the duration of tonic immobility was measured. In cases where tonic immobility did not occur another induction attempt was conducted and the number of inductions was recorded. When tonic immobility could not be attained after 5 induction attempts, a score of 0 s was assigned. When the hen did not stand up within 300 s, the test was stopped and a maximum duration of 300 s was allocated. The observer was out of the hen’s sight during the test. This manipulation induces a reversible catatonic state, the duration of which is positively correlated with general underlying fearfulness [44].

### Eggs

#### Sensory evaluation

As mentioned above, human olfactory/gustatory abilities are commonly used to assess the sensory characteristics of eggs. In a previous study, 2% of menhaden oil was shown to confer a fishy smell to the eggs of Leghorn hens [37]. This perception was corroborated by chromatographic analysis [38]. Such analysis of egg yolks (which contain a high concentration of lipids) is difficult to perform, and therefore we constituted a human panel to assess whether the treatment conferred a fishy smell on the egg yolk. Our panel comprised 14 persons (6 men and 8 women). The formation of individual yolks takes 3 weeks, and therefore a single egg was collected from each hen after 23 days of treatment [45]. Most of the volatile compounds enter the yolk [46], and therefore the yolk alone was extracted, placed in a numbered vial, and frozen (*n* = 19 for control yolks; *n* = 20 for treatment yolks). Each panelist was initially asked to smell a control tube containing 1 mL of menhaden oil. The panelist was then free to randomly pick up and smell each of the 39 vials. For each vial, the panelist recorded whether or not the menhaden oil was detected. The number of panelists who detected menhaden oil odor in each vial was recorded.

#### Incubation

The hens were fertilized by artificial insemination on the 6th day before egg collection, and then once per week during the following 2 weeks. For each group, eggs were collected daily over a period of 9 days and stored at 17°C for incubation. Each female produced 6.3±0.2 eggs, and a total of 250 eggs were collected (125 control eggs and 125 eggs). Eggs of both groups were chosen to obtain no significant difference in egg mass (control eggs, 54.4±0.3 g; treatment eggs, 55.2±0.3 g; ANOVA F_1.248_ = 2.17, *P* = 0.14). The eggs were placed in alternative rows on each shelf of the incubator and maintained at 37.8°C and 56% relative humidity with automatic and continuous turning. On day 7 and day 14 of incubation, non-fertile eggs, or eggs containing prematurely dead embryos were eliminated. Three days before hatching, the rotation was stopped and the temperature was reduced to 37.6°C. Eggs were then placed on a grid constructed of wire mesh and cardboard dividers, to enable identification of control and treatment chicks.

### Chicks

#### Housing conditions

We kept 96 chicks (48 controls and 48 treatment), all hatched on the 21^st^ day of incubation. Each chick was identified with a numbered ring on its leg. The 96 chicks were placed in pairs in wire-covered plastic tubs (50 cm × 40 cm × 30 cm; length × width × height) with wood shavings on the floor, and separated into 2 groups. The pairs of chicks were equally allocated to 2 rooms. Each group was maintained in a 11-h light/13-h dark cycle, with water available *ad libitum*. The chicks were fed *ad libitum* with a conventional starter mash (PEAT, INRA Val de Loire, France) dispensed in feeding troughs (lengh 50 cm). The troughs were covered with a metallic roof containing 12 circular holes (diameter, 5 cm); these holes provided chicks with sufficient access to the feed, while avoiding food spillage. Two opaque drinking bottles (1L) with pipettes were placed in each cage. The ambient temperature was maintained at 33±1.0°C from hatching until chicks were 8 days old, after which it was decreased by 1°C per day to 21±1°C, which was reached when the chicks were 25 days old. The sex of each chick was determined by observation of the comb at 3 weeks of age. The control group was composed of 23 females and 25 males, while the treatment group was composed of 26 females and 22 males.

#### Morpho-physiological measures

The chicks were weighed at hatching, and when they were 9 days, 15 days, 22 days and 29 days old. Their daily (24 h) feed intake was recorded once per week during 5 weeks. The weight of each trough was recorded at 24-h intervals to determine the intake of each pair of chicks.

#### Analysis of feeding preferences

All of the tests used were previously described by Bertin et al. [31], [32]. Chicks become extremely distressed when isolated, and therefore we simultaneously tested 2 chicks from the same exposure conditions in all of the choice tests. Chicks have a very rapid growth rate, and therefore each test was conducted at the same age. This design was also chosen in order to control for the time elapsed between the *in ovo* olfactory stimulation and the test and for the time during which chicks were in contact with their familiar standard food before each test. We conducted 2 types of feeding tests. Firstly a short-term 3-min food-choice test to assess the immediate reaction to novel foods. We used this duration, because previous studies have shown that, under laboratory conditions, chicks have a neophobic reaction lasting around 3 min [44]. In addition, chicks need at least 10 min to associate a specific olfactory cue with a food item [47]. We therefore assumed that control birds were not totally familiar with the olfactory stimulus after the first 3 min of exposure. We also conducted a long-term 24-h food-choice test to analyze feeding preferences and food conservatism (defined as a prolonged reluctance to incorporate novel foods in the diet [48]). Birds exhibit neophobic responses when a single sensorial property of their food is changed [44], [49]. Moreover, the amplitude of neophobic responses is enhanced when multiple sensorial properties of food are changed simultaneously. For example, the visual and tactile properties of food interact, and potentiate the reaction of animals towards novel odors [50]. Therefore, we tested the effect of treatment when chicks were exposed to a change in the olfactory properties of their familiar food (single sensorial modality), and a change in the multiple sensorial properties of the food (unfamiliar food).

The 3-min choice test with menhaden and familiar food: This test was carried out at 4 days of age. The aim was to investigate whether prenatal olfactory experience could modify the reaction of chicks to changes in the olfactory properties of their familiar food (odorized with the olfactory stimulus), and to assess their preference for the familiar food and the odorized familiar food. The testing box, identical to the home box, was located in a different room. The floor was covered with wood shavings and contained 2 feeding troughs (length 50 cm) located at opposite sides. These troughs were identical to the familiar trough, and were similarly covered with a metallic roof containing 12 circular holes (diameter 5 cm); these holes provided chicks with sufficient access to the feed, while avoiding food spillage. One trough contained the familiar starter mash, while the other trough contained the odorized familiar starter mash. Immediately before testing, 0.002 L of menhaden oil was mixed with 1 kg of starter mash; this method enabled the food to be odorized without changing the visual and tactile characteristics. Next, 100 g of the starter mash was placed in one of the feeding troughs, while 100 g of the odorized starter mash was put in the other feeding trough. The location of the troughs was counterbalanced across testing trials. Each pair of chicks was placed in the testing box after 1 h 30 min of food deprivation. The chicks were transported in a 15 cm × 15 cm × 15 cm container, and then deposited and held blind in an enclosure (20 cm × 6 cm × 20 cm) placed equidistant from the 2 troughs. After 30 s, an observer hidden behind a curtain with small observation windows, released and recorded the behavior of 1 focal bird of each pair (24 control birds, 24 treatment birds), for a 3-min period. Focal birds were chosen randomly beforehand, and identified by a blue-colored mark placed on the head. The experimenter recorded the number of distress calls, the latency to explore the food (the bird touched the food with its beak without ingesting it), latency to eat the food (the bird was considered as eating when mandibulation, and neck and throat movements caused by swallowing were observed), time spent eating each food, and number of feeding sequences initiated on each trough (uninterrupted sequence of eating).

The 3-min choice test with menhaden and unfamiliar food: This test was performed at 6 days of age. The aim was to investigate whether prenatal exposure to menhaden odor could modify the reactions of chicks to an unfamiliar food bearing the menhaden odor. The test procedure and variables recorded were similar to those of the 3-min choice test with menhaden and familiar food. However, one trough contained cracked corn-wheat (an unfamiliar food for all groups), while the other trough contained cracked corn-wheat plus the olfactory stimulus (menhaden oil), which was incorporated in the food as described above. The experimenter recorded the behavior of the same focal birds as in the previous test, for a 3- min period.

The 3-min choice test with a novel odor and the familiar food: This test was performed at 13 days of age. The aim was to assess whether the reaction of treatment chicks was generalized to all olfactory stimuli, or specific to the odor of the maternal diet. The test procedure and variables recorded were similar to those of the 3-min choice test with menhaden and familiar food, and the 3-min choice test with menhaden and unfamiliar food. However, one trough contained the familiar starter mash while the other trough contained the odorized familiar starter mash with a translucent powder of isoamyl acetate (0.25 g of powder for 1 kg of starter mash). The experimenter recorded the behavior of the same focal birds, for a 3-min period.

The 24-h choice test with familiar food: This test was carried out at 9–10 days of age. The aim was to investigate whether prenatal olfactory exposure could modify feed preferences and feed intake over a longer time span (24 h). In their home cage, each pair of chicks was provided with a choice between the familiar starter mash, and the starter mash plus the olfactory stimulus (using the same method as in the 3-min choice test). Two familiar troughs were placed on 2 sides of the box for 24 h. The location of the troughs was balanced across pairs of chicks. The weight of each trough was recorded at 24-h intervals to determine the daily feed intake of each pair of chicks.

The 24-h choice test with unfamiliar food: This test was carried out at chicks at 15–16 days of age. The procedure was similar to that of the 24-h choice test with familiar food. However, one trough contained mashed cracked corn-wheat, while the other trough contained mashed cracked corn-wheat plus the prenatal olfactory stimulus.

#### Tonic immobility test

We conducted a tonic immobility test on all 8-day-old chicks. The procedure was the same as that described for adult hens. The number of inductions and the duration of tonic immobility were recorded.

### Data Analysis

We used Kolmogorov-Smirnov tests to determine whether the data were normally distributed. When the distribution did not fit a normal distribution, we used non-parametric statistics. In adult hens, all morpho-physiological measurements were analyzed using one-way ANOVA for repeated measures (treatment × time), and paired *t*-tests for intra-group comparisons. For the sensory evaluation of yolk from control and treatment eggs, we used a permutation test on the total number of panelists who detected menhaden odor for each vial. Fertility and hatching success were tested by using a Chi-square test. We used Mann-Whitney U-tests to compare the laying rate between groups, and performed one-way ANOVAs for repeated measures (treatment × time) to compare the mass of eggs between groups. Variables recorded in the tonic immobility test were analyzed with Mann-Whitney U-tests.

The mass of chicks recorded at 9 days, 15 days, 22 days and 29 days of age was analyzed using ANOVA for repeated measures with treatment and sex as factors. To analyze the effect of treatment in the choice tests, we used a method previously described by Bertin et al. [31], [32]. Raw latencies in scores were transformed, because of their dependence. Latencies to ingest each type of food were converted to latency scores by using the following formula: latency to eat odorized food minus latency to eat non-odorized food. Thus, negative scores indicated that the birds more quickly touched the odorized food than the non-odorized food. Raw durations of time spent eating were also converted. The time spent eating the odorized food was converted into the proportion of time spent eating (time spent eating the odorized food divided by total time spent eating during testing). We performed ANOVA on latency scores, the proportion of time spent eating the odorized food, and the total time spent eating (time spent eating the odorized food plus time spent eating the non-odorized food). We used Mann-Whitney U-tests to compare the number of distress calls emitted by control and treatment chicks during tests. In the 24-h choice tests, we used ANOVAs on the proportion of odorized food eaten (quantity of odorized food eaten divided by total quantity of food eaten).

In addition to the effect of treatment, we analyzed the preferences within each group, by performing intra-group comparisons with paired *t-*tests on the following parameters: latencies to explore and to eat each type of food; number of feeding sequences expressed on each type of food; and time spent eating each type of food. Paired *t-*tests on the raw quantities of each food eaten were used to assess the preferences within each group of chicks in the 24-h choice tests. Data are presented as mean ± SEM. All analyses were performed using Statview software (SAS, Cary, NC), with significance accepted at *P*≤0.05.

## Results

### Laying Females and Eggs

#### Morpho-physiological parameters

ANOVAs on repeated measures revealed no effect of treatment on the mass of hens (ANOVA, F_1.38_ = 0.003, *P* = 0.95). There was a significant effect of time (ANOVA, F_5.38_ = 4.11, *P*<0.001), but no interaction between time and treatment (ANOVA, F_5.190_ = 1.13, *P* = 0.34). Hens in the control and treatment groups were lighter during week 2 than before the start of the treatment (paired *t*-test, *P*<0.05; Table 1). For both group, the mass recorded during the following weeks did not differ significantly from that determined before the start of the treatment (Table 1). We determined no effect of treatment on the daily feed intake (ANOVA, F_1.33_ = 1.02, *P* = 0.32). There was a significant effect of time, but no interaction between time and treatment (ANOVA, time effect: F_5.33_ = 14.9, *P*<0.001; treatment × time: F_5.165_ = 0.37, *P* = 0.87; Table 1).

**Table 1:** Morpho-physiological measurements of adult hens before and during treatment.

Parameters	Hens	Before treatment	Week 1	Week 2	Week 3	Week 4	Week 5
Mass (kg)	MH	1.75±0.03	1.72±0.03	1.68±0.04 *	1.71±0.04	1.72±0.04	1.72±0.04
	Controls	1.73±0.04	1.71±0.04	1.70±0.04 *	1.72±0.04	1.72±0.04	1.73±0.04
Daily feed Intake (g)	MH	158±26.2	66.6±6.86*	93.5±9.30*	83.6±7.57*	78.9±6.10*	90.44±3.61*
	Controls	179±28.4	81±8.94*	88.2±6.86*	76.4±6.02*	93.1±5.85*	94.2±5.88*
Laying rate (numberper female per day)	MH	0.77±0.05	0.76±0.05	0.80±0.03	0.74±0.06	0.77±0.05	0.79±0.03
	Controls	0.75±0.06	0.80±0.03	0.86±0.03	0.83±0.04	0.82±0.03	0.81±0.04
Mass of eggs (g)	MH	54.3±0.72	54.1±0.76	53.9±0.84	54.8±0.88	54.6±0.86	54.5±0.76
	Controls	54.9±0.85	55.2±0.87	55.7±0.91*	55.6±0.94	55.7±0.96 *	56.3±0.89 **

All data are represented as mean ± SEM (*n = *20). Paired *t-*test (comparison with “before treatment”):

* *P*<0.05,

** *P*<0.01.

#### Laying rate and mass of eggs

We determined no significant effect of treatment on the laying rate (Mann-Whitney, *n_1_* = 20, *n_2_* = 20, *z* = −1.77, *P* = 0.07) or mass of eggs (ANOVA, F_1.36_ = 0.67, *P* = 0.42). There was an effect of time (ANOVA, F_5.36_ = 2.87, *P*<0.05), and a significant interaction between time and treatment (ANOVA, F_5.180_ = 3.53, *P*<0.01). During weeks 4 and 5, control eggs were heavier than before treatment started; by contrast, the mass of treatment eggs remained stable (Table 1).

#### Olfactory evaluation of eggs

We observed a large variability between vials within each group (Table 2). The number of panelists detecting menhaden oil was significantly higher for the group of vials containing treatment yolks than for the group of vials containing control yolks (permutation test, *P*<0.001; Table 2).

**Table 2:** Olfactory evaluation of egg yolks.

Treatment yolks	Number of panelists	Control yolks	Number of panelists
MH1	9	C1	0
MH2	9	C2	7
MH3	10	C3	4
MH4	1	C4	8
MH5	5	C5	2
MH6	11	C6	7
MH7	3	C7	3
MH8	4	C8	0
MH9	7	C9	1
MH10	11	C10	2
MH11	8	C11	6
MH12	7	C12	3
MH13	14	C13	3
MH14	12	C14	4
MH15	3	C15	3
MH16	4	C16	6
MH17	10	C17	11
MH18	3	C18	6
MH19	6	C19	5
		C20	8

Data represent the number of panelists who detected menhaden odor in each vial.

#### Tonic immobility tests

We determined no significant effect of treatment on tonic immobility duration, or on the number of induction attempts (number of inductions: controls vs. MH hens: 2.05±0.32 vs. 1.85±0.26; Mann-Whitney, *n_1_* = 20, *n_2_* = 20, *z* = −0.38, *P* = 0.73; duration: controls vs. MH hens: 71.3±19.0 s vs. 75.1±16.4 s; Mann-Whitney, *n_1_* = 20, *n_2_* = 20, *z* = −0.37, *P* = 0.71).

### Chicks

#### Morpho-physiological measures

The hatching rate did not differ significantly between the treatment and control groups (controls, 84.8%; MH chicks, 75.4%; Chi-square, *P* = 0.55). We determined no significant effect of treatment on the mass of chicks (ANOVA, F_1.92_ = 0.78, *P* = 0.38). There was an effect of time, but no interaction between time and treatment (ANOVA, time effect: F_4.92_ = 18912, *P*<0.001; treatment × time: F_4.368_ = 0.42, *P* = 0.79; Table 3). We determined a significant effect of sex (sex effect: F_1.92_ = 76.6, *P*<0.001; sex × time: F_4.368_ = 108, *P*<0.001). In treatment and control groups, male chicks were heavier than females and grew faster.

**Table 3:** Growth and feed intake of chicks.

Parameters	Chicks	Week 1	Week 2	Week 3	Week 4	Week 5
Mass (g)	MH	38.5±0.50^a^	75.0±0.83^b^	144±1.92^c^	240±3.18^d^	357±5.09^e^
	Controls	39.7±0.50^a^	76.4±0.70^b^	145±1.49^c^	244±2.85^d^	363±4.40^e^
Daily feed Intake (g)	MH	11.5±0.51^a^	20.79±0.98^b^		58.54±1.24^c^	75.75±1.65^d^
	Controls	10.92±0.38^a^	20.28±1.10^b^		59.21±1.70^c^	80.54±2.07^d^

All data are represented as Means ± SEM mass of MHchicks (n = 48) and control chicks (n = 48) and daily intake measured in pairs of chicks. Different superscript letters indicate significant differences in paired *t-*tests (*P*<0.05).

We determined no significant effect of treatment on the daily feed intake (ANOVA, F_1.46_ = 1.30, *P* = 0.26). There was an effect of time, but no interaction between time and treatment (ANOVA, time effect: F_3.46_ = 1198, *P*<0.001; treatment×time: F_3.138_ = 1.86, *P* = 0.14; Table 3).

#### The 3-min choice test with menhaden and familiar food

Between groups comparisons revealed no significant differences (latency score to explore: controls = −34.7±28.1 s vs. MH chicks = −20.0±25.8 s; ANOVA, F_1.46_ = 0.15, *P* = 0.70; latency score to eat: controls = −26.4±29.2 s vs. MH chicks = −17.9±25.4 s; ANOVA, F_1.45_ = 0.04, *P* = 0.85; proportion of time spent eating the odorized food: controls = 0.58±0.1 vs. MH chicks = 0.54±0.1; ANOVA, F_1.45_ = 0.05, *P* = 0.82; total time spent eating: controls = 67.1±33.6 s vs. MH chicks = 60.5±31.0 s, ANOVA, F_1.46_ = 0.49, *P* = 0.49; number of distress calls: controls = 5.92±5.01 vs. MH chicks = 14.6±10.2; Mann-Whitney, *n_1_* = 24, *n_2_* = 24; *z* = 0.40, *P* = 0.69).

Within group comparisons revealed no behavioral difference according to the type of food (controls: latency to explore the odorized food vs. latency to explore the non-odorized food = 77.4±15.1 s vs. 112±15.5 s; paired *t*-test, *t* = −1.23, *P* = 0.23; latency to eat the odorized food vs. latency to eat the non-odorized food = 86.7±15.4 s vs. 113±15.3 s, *t* = −0.91, *P* = 0.38; time spent eating the odorized food vs. time spent eating the non-odorized food: 35.3±7.6 s vs. 31.8±9.2 s, *t* = 0.74, *P* = 0.4; number of feeding sequences on odorized food vs. number of feeding sequences on non-odorized food = 5.33±1.1 vs. 4.33±1.2, *t* = 0.47, *P* = 0.65; MH chicks: latency to explore the odorized food vs. latency to explore the non-odorized food = 83.3±14.0 s vs. 103±15.1 s, paired *t*-test, *t* = −0.77, *P* = 0.45; latency to eat the odorized food vs. latency to eat the non-odorized food = 87.2±13.8 s vs. 105±14.8 s, *t* = −0.70, *P* = 0.49; time spent eating the odorized food vs. time spent eating the non-odorized food = 31.6±6.8 s vs. 29.0±8.1 s, *t* = 0.44, *P* = 0.66; number of feeding sequences on odorized food vs. number of feeding sequences on non-odorized food = 4.58±0.92 vs. 3.67±0.93, *t* = 0.53, *P* = 0.60).

#### The 3-min choice test with menhaden and unfamiliar food

**Figure 1:**
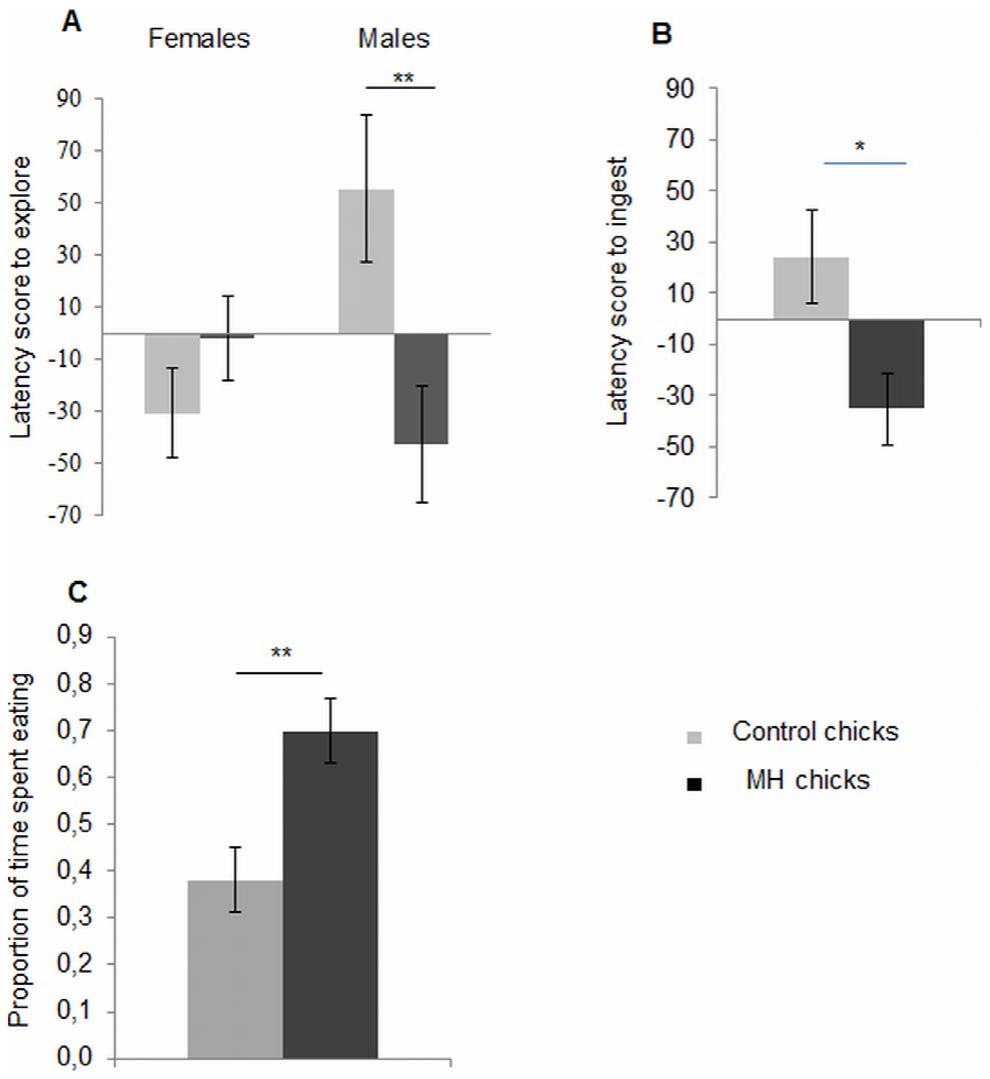
Comparison of the behavior of control (*n* = 24 pairs) and MH chicks (*n = *24 pairs) in the 3-min choice test with menhaden and unfamiliar food. (A) Mean ± SEM latency score to explore the odorized unfamiliar food. (B) Mean ± SEM latency score to ingest the odorized unfamiliar food. (C) Mean ± SEM proportion of time spent eating the odorized unfamiliar food. ANOVA, *P<0.05.

MH chicks tended to explore menhaden-odorized unfamiliar food faster than did control chicks (controls = 18.48±18.86 s vs. MH chicks = −25.79±15.02 s; ANOVA, F_1.47_ = 3.34, *P* = 0.07). There was no significant effect of sex (sex effect: ANOVA, F_1.44_ = 1.03, *P* = 0.31). However, there was a significant interaction between sex and treatment (sex × treatment: ANOVA, F_1.44_ = 8.06, *P*<0.01). MH males explored the odorized unfamiliar food significantly faster than did control males (ANOVA, F_1.24_ = 7.58, *P*≤0.05). This effect was not observed in females (ANOVA, F_1.20_ = 1.42, *P* = 0.25; Fig. 1A). MH chicks ingested the odorized unfamiliar food significantly sooner than did control chicks (ANOVA, F_1.47_ = 6.53, *P*<0.05; Fig. 1B). We determined no global effect of sex on the latency score to ingest (ANOVA, F_1.44_ = 0.35, *P* = 0.56). There was an almost significant interaction between sex and treatment (ANOVA, F_1.44_ = 3.72, *P* = 0.06). Control males took longer to ingest the odorized unfamiliar food than did MH males (control males = 46.9±25.9 s vs. MH males = −47.9±18.6 s; ANOVA, F_1.24_ = 9.21, *P*≤0.01). This effect was not present in females (control females = −10.2±23.3 s vs. MH females = −17.6±22.1 s; ANOVA, F_1.20_ = 0.05, *P* = 0.82). The proportion of time spent eating the menhaden-odorized unfamiliar food was significantly higher in MH chicks than in control chicks (ANOVA, F_1.47_ = 12.15, *P*<0.001; Fig. 1C). The total time spent eating during testing was lower in MH chicks than in control chicks (controls = 59.4±8.68 s vs. MH chicks = 36.3±3.99 s; ANOVA, F_1.47_ = 6.93, *P*<0.05). MH chicks emitted significantly more distress calls than did control chicks (controls = 0±0 vs. MH chicks = 6.46±3.20, *n_1_* = 24, *n_2_* = 24; Mann-Whitney, *z* = −1.50, *P*<0.01).

**Figure 2:**
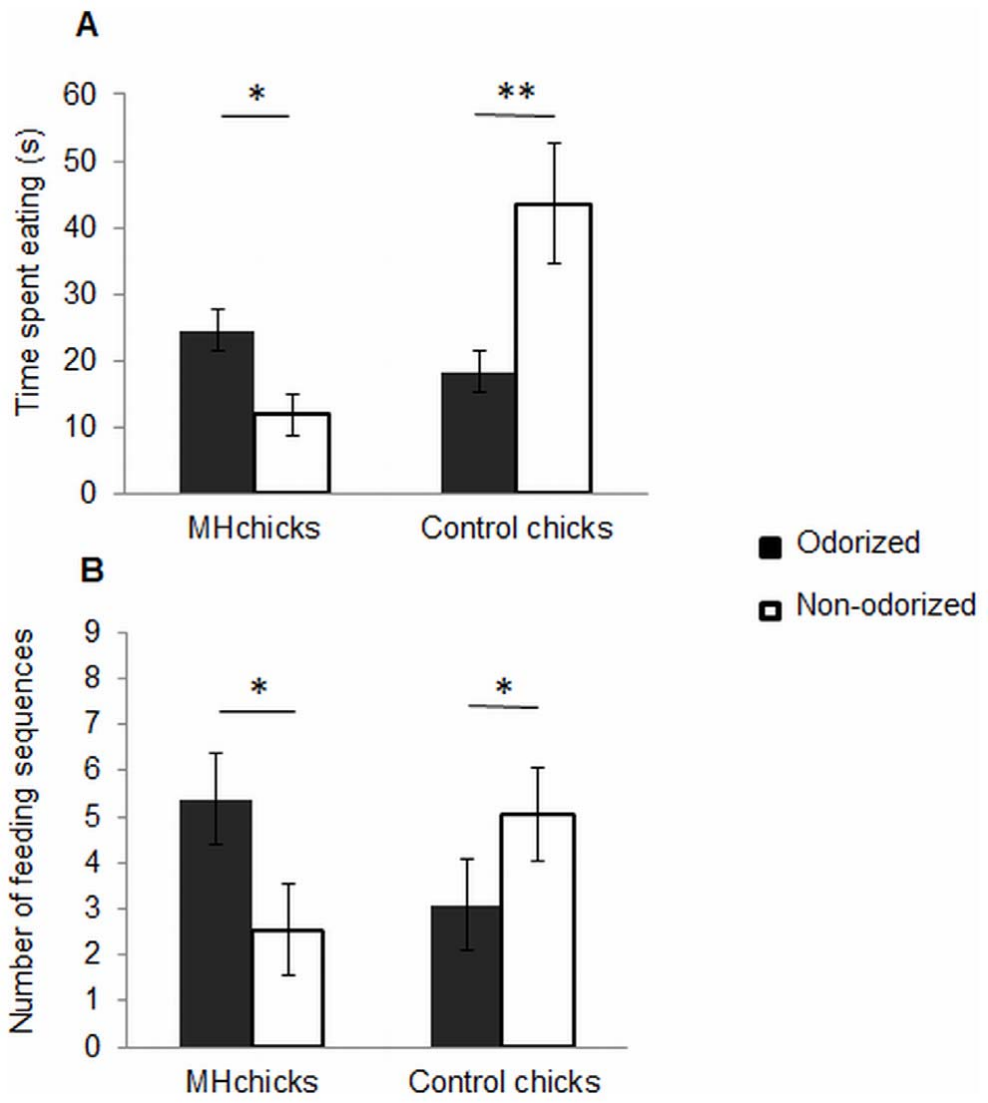
Preferences within control and MH chicks for the odorized or non-odorized unfamiliar food. (A) Mean ± SEM time spent eating each type of food. (B) Mean ± SEM number of feeding sequences on each type of food. Paired *t-*test, *P<0.05, **P<0.01.

Within the MH group, chicks ingested the odorized unfamiliar food significantly sooner than the non-odorized unfamiliar food (30.5±4.6 s vs. 65.8±12.2 s, paired *t*-test, *t* = −2.4, *P*<0.05). MH chicks also spent significantly more time eating the odorized unfamiliar food than the non-odorized unfamiliar food (paired *t*-test, *t* = 2.11, *P*<0.05; Fig. 2A). Furthermore, MH chicks expressed significantly more feeding sequences on the odorized unfamiliar food than on the non-odorized unfamiliar food (paired *t*-test, *t* = 2.36, *P*≤0.05; Fig. 2B). Within the control group, the latencies to ingest the odorized unfamiliar food and the non-odorized unfamiliar food did not differ significantly (62.1±13.8 s vs. 37.8±8.4 s, paired *t*-test, *t* = 1.3, *P* = 0.2). Control chicks spent significantly less time eating the odorized unfamiliar food than the non-odorized unfamiliar food (paired *t*-test, *t* = −2.87, *P*<0.01; Fig. 2A). However, the numbers of feeding sequences expressed on each type of food did not differ significantly (paired *t*-test, *t* = −2.38, *P*≤0.05; Fig. 2B).

#### The 3-min choice test with a novel odor and the familiar food

Between groups comparisons revealed no significant differences (latency score to explore: controls = 13.0±24.7 s vs. MH chicks = −17.0±21.8 s; ANOVA, F_1.46_ = 0.83, *P* = 0.37; latency score to eat: controls = 15.5±24.3 s vs. MH chicks = −0.58±22.13 s; ANOVA, F_1.46_ = 0.24, *P* = 0.63; proportion of time spent eating the odorized familiar food: controls: 0.38±0.09 vs. MH chicks = 0.51±0.09; ANOVA, F_1.46_ = 1.02, *P* = 0.32; total time spent eating during testing: controls: 46.50±7.41 vs. 49.87±7.91; ANOVA, F_1.46_ = 0.10, *P* = 0.76; number of distress calls: controls = 0.08±0.08 vs. MH chicks = 1.04±0.65, Mann-Whitney, *n_1_* = 24, *n_2_* = 24; *z* = −1.10, *P* = 0.27).

Within group comparisons revealed no behavioral difference according to the type of food (controls: latency to explore the odorized food vs. latency to explore the non-odorized food = 75.6±15.6 s vs. 54.4±11.7 s, *t* = 0.89, *P* = 0.38; latency to eat the odorized food vs. latency to eat the non-odorized food = 78.1±15.4 s vs. 62.6±12.3 s, *t* = 0.64, *P* = 0.53; time spent eating the odorized food vs. time spent eating the non-odorized food = 16.8±6.0 s vs. 33.0±7.6 s, *t* = −1.45, *P* = 0.16; number of feeding sequences on odorized food vs. number of feeding sequences on non-odorized food = 2.58±0.8 vs. 3.38±0.6, *t* = −0.63, *P* = 0.53; MH chicks: latency to explore the odorized food vs. latency to explore the non-odorized food = 66.0±13.6 s vs. 76.4±13.1 s, paired *t*-test, *t* = −0.50, *P* = 0.62; latency to eat the odorized food vs. latency to eat the non-odorized food = 82.3±14.4 s vs. 82.9±13.8 s, *t* = −0.03, *P* = 0.98; time spent eating the odorized food vs. time spent eating the non-odorized food = 17.0±4.6 s vs. 29.5±7.4 s, *t* = −1.27, *P* = 0.22; number of feeding sequences on odorized food vs. number of feeding sequences on non-odorized food = 2.4±0.59 vs. 3.3±0.84, *t* = −0.79, *P* = 0.44).

#### Comparison of the total time spent eating during the 3 tests

**Figure 3:**
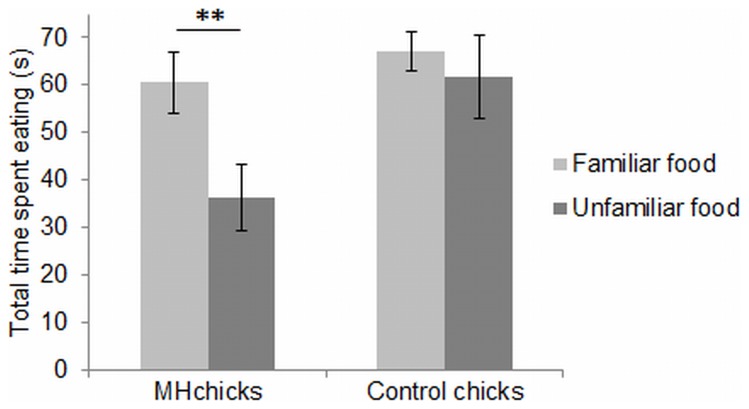
Comparison of the total time spent eating during the 3-min choice test with menhaden and the familiar food, and the 3-min choice test with menhaden and the unfamiliar food. Paired *t-*test, **P<0.01.

MH chicks spent significantly less total time eating during the 3-min choice test with menhaden and unfamiliar food than during the 3-min choice test with menhaden and familiar food (36.3±19.5 s vs. 60.5±31.0 s; paired *t*-test, *t* = 3.47, *P*<0.01). For control chicks, the total time spent eating did not differ significantly between the 2 tests (59.4±42.5 s vs. 67.1±33.6 s; paired *t*-test, *t* = 0.72, *P* = 0.48; Fig. 3). The total time spent eating did not differ significantly between the 3-min choice test with menhaden and familiar food and the 3-min choice test with a novel odor and the familiar food, either in MH chicks (paired *t*-test, *t* = 1.41, *P* = 0.17) or in control chicks (paired *t*-test, *t* = 1.54, *P* = 0.14).

#### The 24–h choice test with familiar food

The quantity of familiar odorized food eaten did not differ significantly between groups (ANOVA, F_1.46_ = 0.89, *P* = 0.35). The quantity of odorized or non-odorized food eaten did not differ significantly, either in control chicks (21.3±2.32 g vs. 20.2±2.15 g; paired *t*-test, *t* = −0.27, *P* = 0.79) or in MH chicks (25.0±2.60 g vs. 20.0±2.60 g; *t* = −1.04, *P* = 0.31).

#### The 24–h choice test with unfamiliar food

The quantity of unfamiliar odorized food eaten did not differ significantly between groups (ANOVA, F_1,47_ = 0.11, *P* = 0.74). Control chicks ingested a significantly higher quantity of odorized unfamiliar food than non-odorized unfamiliar food (19.9±2.44 g vs. 11.9±1.67 g; paired *t*-test, *t* = −2.35, *P*<0.05). MH chicks tended to eat more menhaden-odorized unfamiliar food than non-odorized unfamiliar food (19.93±2.31 g vs. 12.6±1.60 g; paired *t*-test, *t* = −1.91, *P* = 0.07).

#### Tonic immobility test

**Figure 4:**
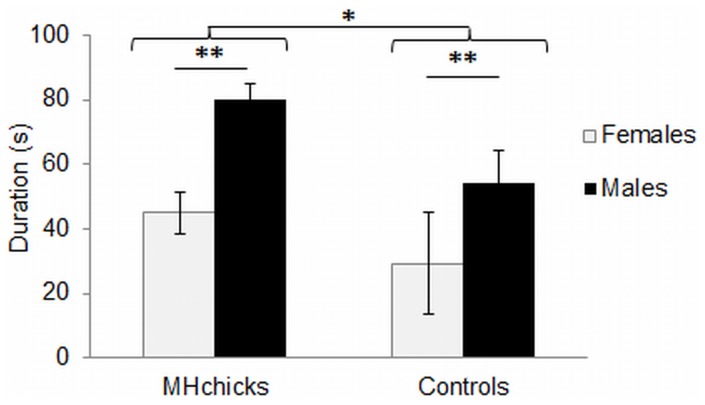
Mean ± SEM tonic immobility duration (s) in chicks. MH chicks (*n = *48) and control chicks (*n* = 48); ANOVA, *P<0.05, **P<0.01.

The number of inductions did not differ significantly between groups (controls = 1.54±0.16; MH chicks = 1.88±0.19; ANOVA, treatment: F_1.92_ = 1.58, *P* = 0.21; sex: F_1.92_ = 1.71, *P* = 0.19; treatment × sex: F_1.92_ = 0.04, *P* = 0.84). MH chicks had a significantly longer duration of immobility than did control chicks (ANOVA, F_1.92_ = 4.28, *P*<0.05). There was a significant effect of sex (ANOVA, F_1.92_ = 9.04, *P*<0.01), but no significant interaction between sex and treatment (ANOVA, F_1.92_ = 0.26, *P* = 0.61). Irrespective of treatment, males showed higher durations of TI than did females (Fig. 4).

## Discussion

The results of the present study contribute to our understanding of non-genetic maternal influences on the development and phenotype outcome of offspring. The main results can be summarized as follows: (i) offspring of hens fed with a diet containing menhaden oil showed a short-term preference for unfamiliar food bearing the scent of menhaden; and (ii) when confronted with a novel food or restraint in a tonic immobility test, offspring of hens fed with a diet containing menhaden oil expressed stronger fear reactions than did control chicks. Our data indicate for the first time that subtle changes in oil composition of the maternal diet may engender maternal effects, and orient the behavioral phenotype of young birds.

### Effect of Treatment on the Parental Population and Eggs

We determined no significant effect of the addition of 2% menhaden oil in the diet on the behavior and morpho-physiological parameters of hens. Independent of treatment, we observed a decrease in feed intake at the beginning of the experimental period, which may have been caused by the transition from a flour diet to granules. The changes in the characteristic of the food (form, odor, texture) may have engendered a transitory neophobic reaction, which is commonly observed in poultry [44], [48]. However, contrary to flours, granules are more concentrated in essential nutrients, and therefore do not need to be consumed in great quantity. The increase in the mass of eggs laid by control hens may have been caused by the presence of soybean oil in the granules [51]. To counterbalance a potential effect of egg size on the developmental trajectory of offspring, we equilibrated the mass of eggs placed in incubators. Therefore, we believe that this parameter was unlikely to be responsible for the behavioral differences observed in chicks.

Regarding the transmission of olfactory compounds from the maternal diet to the egg yolk, several studies have shown that fatty acids, including menhaden oil, confer a “fishy” smell to the yolk [35], [36], [37], [46], [52]. In the present study, the menhaden oil scent was more frequently identified by humans in treatment vials than in control vials, although there was high variability between vials. Boiling and tasting yolk (a classic method which involves the use of olfactory and gustatory perceptual systems) might have been a better method for enhancing the detection threshold; however it would have required a much higher number of samples and would therefore have reduced the number of eggs available for incubation.

### Effect of Treatment on the Short-term Feeding Preferences of Offspring

The higher detection scores of menhaden scent in the treatment vials compared to the control vials, coupled with the clear behavioral differences observed in the 3-min choice test with menhaden and unfamiliar food, lead us to believe that MH chicks were influenced by the menhaden scent *in ovo*. When confronted with the unfamiliar food bearing menhaden oil, male MH chicks explored this food significantly faster than did male control chicks. MH chicks also ingested the food sooner, and spent a higher proportion of time eating it, than did control chicks. In addition, intra group comparisons revealed opposite food preferences between MH and control chicks. MH chicks ingested the odorized unfamiliar food sooner than the non-odorized unfamiliar food. In addition, they spent a higher percentage of time eating it, and expressed a higher number of feeding sequences on it. These clear differences indicate a positive orientation towards the unfamiliar food bearing the olfactory stimulus, which is a classic trait observed in animals exposed to a scent *in utero* or *in ovo* [4], [9], [14], [19]. This orientation towards the food bearing the menhaden scent was not observed in control chicks, which spent more time eating the non-odorized unfamiliar food than the odorized unfamiliar food. The unfamiliar odor associated with the novel visual characteristics of the food may have induced avoidance behavior in control chicks. This response towards cumulative changes in the sensorial characteristics of food has frequently been observed in poultry [44], [53]. On the other hand, the positive orientation of MH chicks towards the menhaden oil may be explained by the capacity of chicks to use *in ovo* chemosensory memory to orient their feeding behavior. Information regarding the maternal diet acquired *in ovo* may help young precocial birds to identify and consume palatable food in their environment. Normally, precocial chicks benefit from the experience of their mothers to select food items, and preferences are transmitted from mothers to chicks [54]. However, precocial birds also learn by themselves whether food is edible, by pecking at a large range of items during the first days of their lives [55]. According to the transnatal olfactory continuity hypothesis [39], *in ovo* perceptual learning can facilitate this training, by orientating early pecking behavior towards familiar sensorial characteristics.

We determined no effect of treatment in the 3-min choice test with familiar food and menhaden oil, or in the 3-min choice test with a novel odor and familiar food. Within each group of chicks, we observed no preference for odorized or non-odorized food. Our results contradict previous data obtained using domestic chicks [30], [31], [32], [34], [53], [56]. Commonly, control birds show a transitory or consistent avoidance of food (or water) odorized with an unfamiliar scent. In the present study, the classical neophobic reaction observed in chicks was not present in control chicks or MH chicks confronted with the unknown isoamyl acetate scent. One possible explanation may lie in the different lines of hens used. All previous studies were conducted with broilers, whereas in the present study we used laying hens. In laying hens, the visual aspect of food may be the principal factor that determines pecking behavior. This may explain why the positive orientation towards the menhaden scent was observed only when the chicks were confronted with a food showing unfamiliar visual characteristics. Our paradigm with familiar food may therefore be inappropriate for elucidating the prehatch sensory experience of laying hens. Chicks were previously shown to express a preference for the first food to which they were exposed [57], [58], from just after hatching until 3 days old [55]. Therefore, our present results may also be explained by a stronger visual “imprinting” in laying hens than in broilers.

### Effect of Treatment on the Long-term Feeding Preferences of Offspring

At 9 days of age, we determined no effect of treatment, and no preference between the familiar food and the familiar food bearing the menhaden scent. On the other hand, at 16 days of age, control chicks ingested a significantly greater quantity of the unfamiliar food bearing the menhaden scent than the unfamiliar non-odorized food. No clear preference was observed in MH chicks. Several authors have demonstrated that chicks are able to select their food according to their nutritional needs [55], [57], [58], [59]. In addition, on a 24-h time scale, domestic hens are able to associate and memorize sensory qualities of food, and subsequent post-ingestive effects [60]. Cereals mixed with oil (albeit in a subtle concentration) may have altered and enhanced the energetic quality compared to non-odorized cereals. Subtle changes in the concentrations of yolk hormones of maternal origin have been shown to influence the physiology and morphology of the chicks [61], [62]; in the same way, it is possible that a subtle difference in the lipids contained in egg yolk can modify the metabolism of chicks. Control chicks may have associated the menhaden scent with a nutritional contribution– particularly omega-3– or energy, corresponding better to their needs. This was not observed with the familiar food, possibly because the strong imprint of first food masked this effect.

We could also not exclude the hypothesis that, at 9–10 days of age, the experience with the familiar food bearing the menhaden scent influenced the preferences observed at 16 days of age. The *in ovo* olfactory experience and posthatch experience with different types of foods probably influence the way in which chicks associate sensorial characteristics of foods with specific post-ingestive consequences [31], [32].

### Effect of Treatment on Food Neophobia and Fearfulness of Offspring

The idea that control chicks and MH chicks differ in their metabolism and nutritional needs may, albeit speculatively, be explained by the clear differences observed in underlying fearfulness. Several parameters indicated that MH chicks were more fearful than control chicks. Firstly, MH chicks comprised the only group in which food neophobia was observed. When confronted with the unfamiliar food, MH chicks spent less than half the time eating than did control chicks. MH chicks also comprised only group to emit distress calls when confronted with the cereals. In addition, whereas control chicks spent equivalent amounts of time eating during the 3-min choice tests with familiar and unfamiliar food, MH chicks spent much less time (less than half the time compared with control chicks) eating cereals. These data clearly show a reaction of food neophobia in MH chicks.

Secondly, MH chicks showed significantly longer durations of tonic immobility than did control chicks. Tonic immobility is a reversible spontaneous catatonic response induced by a constraint, and is considered to be positively correlated with underlying fearfulness in poultry [63], [64]. Taken together, our data indicate a higher level of emotional reactivity in MH chicks than in control chicks. We further revealed an effect of sex, with males showing higher immobility duration than females. This effect has previously been reported in domestic hens [65], [66].

The literature on mammals provides a number of possible explanations for these differences in emotional reactivity. Menhaden oil contains more omega-3 than omega-6 (30% omega-3 vs. <10% omega-6). Soybean oil, although with equivalent energetic value, contains more omega-6 than omega-3 (7% omega-3 vs. 53% omega-6). Fatty-acids such as omega-3 and omega-6 are known to pass from the mother’s diet to the egg yolk [35], [67]; however, the effect of these fatty acids on the offspring have not yet been investigated. In rodents, omega-3 [40] and omega-6 [68] contained in the diet were found to increase anxiety-like behaviors. The addition of omega-3 fatty acids from fish oil to the diet was shown to decrease anxiety in the gray mouse lemur (*Microcebus murinus*) and humans [41], [69]. Long-chain polyunsaturated fatty acids are considered essential for the proper functioning of the mammalian central nervous system. Although further research is required to support our theory, it is possible that the same trait occurs in birds. Eggs containing more or less fatty acids as a consequence of the maternal diet may orient the development of the central nervous system, and thereby the behavior of offspring.

## Conclusions

Our data indicate that subtle changes in oil composition of the maternal diet influence the behavioral development of offspring in birds. Our results have potentially broad implications for the study of maternal effects in laboratory or farm animals, and also for conservation biology, where the effects of parental population management must be monitored on multiple levels. In the feed engineering industry, the composition of the diet provided to breeding hens can vary according to the price of raw materials. It is worth investigating whether certain types of raw material influence the quality of eggs and the subsequent behavior–such as fearfulness–of broiler chickens and laying hens. The potential impact of the maternal diet on food neophobia has considerable ecological implications, because food neophobia can compromise the ability of birds to cope with novel environments and novel food resources [70]. Finally, our data indicate that the chemosensory experience *in ovo* orients subsequent feeding behavior. This suggests a common principle of sensory system development across vertebrate taxa.
